# Hospitalizations in Adults With Alzheimer’s Disease and Related Dementia—United States, 2016–2017

**DOI:** 10.1093/geroni/igaa057.1594

**Published:** 2020-12-16

**Authors:** Christopher Taylor, Benjamin Olivari, Lisa McGuire

**Affiliations:** 1 Centers for Disease Control and Prevention, Atlanta, Georgia, United States; 2 CDC, Atlanta, Georgia, United States

## Abstract

Alzheimer’s disease and related dementias (ADRD) are a significant public health burden. Collectively, ADRD have been called the most expensive chronic conditions in the United States due to outsized health care utilization. Data from the 2016 and 2017 Healthcare Cost Utilization Project National Inpatient Sample, an all-payer representative sample of US hospitalizations, were used to describe hospitalizations in adults ≥65 years with ADRD. Chronic conditions were defined using International Classification of Disease, Tenth Edition, Clinical Modification (ICD-10-CM) code definitions from the Centers for Medicare and Medicaid Services Chronic Conditions Warehouse. Code definitions from the Agency for Healthcare Research and Quality defined potentially preventable hospitalizations where admission might have been avoided by appropriate outpatient primary care management. One in six hospitalizations in adults ≥65 years were for persons with ADRD, including 1 in 3 adults ≥85 years. Among those with ADRD-related admissions, the most common reasons for admission, as defined by the principal diagnosis, were heart disease (18.1%), certain infections (14.5%), injuries (12.7%), respiratory illness (11.2%), and genitourinary conditions (10.4%). In hospitalized adults with ADRD, the prevalence of diagnosed urinary tract infection (37.0%)—a potentially preventable hospitalization—is more than double the prevalence in adults without ADRD (15.5%, prevalence ratio = 2.39, 95% confidence interval: 2.37-2.42). Common comorbidities, injuries, and potentially preventable hospitalizations all contribute to hospitalizations in adults with ADRD. Focusing on injury prevention and appropriate outpatient management of comorbidities in adults with ADRD might reduce the number of hospitalizations, including potentially preventable hospitalizations, among adults with ADRD.

